# Tuberculosis Notes

**Published:** 1920-12-18

**Authors:** 


### TUBERCULOSIS NOTES.

### Children's Balconies.

At the Beckett Hospital, Barnsley, a sum of money amounting to about £1,000, the remainder of a fund collected by the Ladies' War League Committee, has been offered for the purpose of building a children's balcony. This would obviously be best put on the south wall of the building, where the most clement aspect possible would be assured, with, consequently, the greatest possible number of days' use. Further, the modern practice of heliotherapy in surgical tuberculosis clearly dictates a southern exposure too. However, a hitch has arisen in that some of the parties concerned are reported to want a different placing of the proposed addition. These divergences of opinion are always regrettable, and are perhaps peculiarly liable to arise in this country. On the Continent, and in America too, all such points would be adjudged technical matters, to be settled by technical judgment. The interests of patients, obviously the primary consideration, demand that the best professional opinion should be followed, to the exclusion of all other.

### All Schools Open Air?

In a recent conversation the matron of one of these schools deplored the fact that the children must return to over-crowded houses for long hours, and particularly in winter-time, when playing in the streets was impossible. Trams, too, whilst useful because they convey the children to and from school without undue exertion, were condemned because they were "stuffy." Why should open-air schools be only day schools? But if they must be so, why not have open trams, such as one sees on the Continent or motor charabancs? It is a fact that all children lose weight during holidays, and that colds are more frequent during the first few days of the week than during the last two. But until we have peace at home and abroad such schemes must wait to be realised, and so long will children suffer. When conditions improve—and we include under this heading such things as a pure milk supply, good, plentiful food, and fresh, not smoke-polluted, air, houses fit to live in, and leisure to enjoy them, then tuberculosis will diminish and practically disappear. All other measures must, by the way things are moving, be more or less temporary; but they serve a useful purpose, and must be encouraged. Dr. Trimble's comment on the many who do not follow advice unless it be the formula of medicine three times a day is true as far as it goes; but he overlooks in his turn that to exercise personal and household cleanliness and to live an open-air life is a physical impossibility for so many.

### Surgical Measures in Phthisis.

The last special number on lung collapse therapy of the *Centralblatt für die gesamte Tuberkulose-Forschung* has some information which will interest the now numerous body of workers in artificial pneumothorax. Sauerbruch gives his extended results in operative treatment, mainly extra-pleural thoracoplasty—381 cases; mortality 2 per cent. in the first two days, 7 per cent. in first ten days; cures (without expectoration or pyrexia three to four years after the operation) 35 per cent. It must be remembered that these are severe cases of phthisis, on whom artificial pneumothorax has been impossible, or has failed. He remarks of unilateral phrenicotomy that the paralysed half of the diaphragm rises up and compresses the lower lobe, so that it is chiefly applicable to lower-lobe disease. However, it is only a partial and insufficient measure, just like costal resection at the apex only. The two measures need combining, or preferably an upper costal resection with a pneumothorax below.

If there is a pneumothorax below, and one resorts to "plugging" (introducing an aseptic foreign body between rib and lung in order to compress the latter) with the object of compressing an adherent apex, there is the danger that the thin pleura separating the pneumothorax from the plug will not bear the weight of it.

All these surgical measures for lung collapse are, of course, necessitated by artificial pneumothorax being made impossible by total adherence of the two layers of the pleura, or else insufficient by their partial welding. However, elsewhere in the number, Rieckenberg describes another expedient. It is to make, as it were, an extra-pleural pneumothorax; to separate, through a 20 c.m. incision in a middle intercostal space, the outer surface of the costal pleura from the ribs, doing it manually by getting the whole hand into the thoracic cavity. Obviously this will be a difficult process to accomplish fully, and if only done partially the well-known drawbacks to partial compression, already mentioned above, will have a chance to come into play. But as a less severe measure than extra-pleural thoracoplasty, it deserves a trial.

